# Supplementary material for: Cognitive Training Therapy Improves the Effect of Hypocaloric Treatment on Subjects with Overweight/Obesity: A Randomised Clinical Trial
Source: Nutrients. 2019 Apr 24;11(4):925. doi: 10.3390/nu11040925 (PMC6521325; doi:10.3390/nu11040925)

Supplementary information. Figure S1. Baseline performance on the Iowa Gambling task for the five blocks of task execution in both CONTROL and COGNITIVE groups. Mean differences were analyzed by a *t*-test. Significance level is shown in parenthesis.

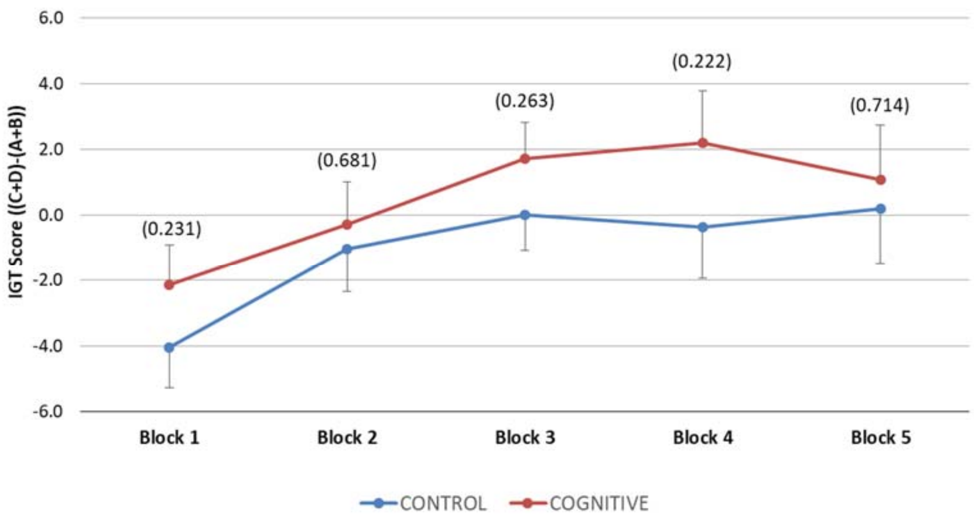

Supplement: Supplementary file 1 [file nutrients-11-00925-s001.zip › Figure S1_IGT blocks.pdf]
