# Supplementary material for: Cognitive Training Therapy Improves the Effect of Hypocaloric Treatment on Subjects with Overweight/Obesity: A Randomised Clinical Trial
Source: Nutrients. 2019 Apr 24;11(4):925. doi: 10.3390/nu11040925 (PMC6521325; doi:10.3390/nu11040925)

Supplementary information SI-1. Neurocognitive Intervention Additional information.

COGNITIVE group participants performed a cognitive training during 12 sessions, carried out during the 3 months of hypocaloric treatment. The training was carried out with the PC game Brain Exercise™ (Bandai Namco Games Ltd.).

Participants held the training sessions on an HP 250 G3 Notebook PC (Windows 10), with a 15-inch screen, and a resolution of 1366x768x40 Hz. The performance achieved by each participant in each session was recorded to analyze the evolution of the subjects. The participants made a total of 12 practice exercises, which implies approximately 30 minutes per session.

Brain Exercise™ game was selected because is one of the most popular memory training games in the world (PC version of the Brain Training game console, Nintendo Co. Ltd.) (Figure S2). In addition, this game was developed with a high level of evidence in neuropsychology and psychology(1,2), and above all, because previous studies have shown its effectiveness to increase certain cognitive functions(3–6).

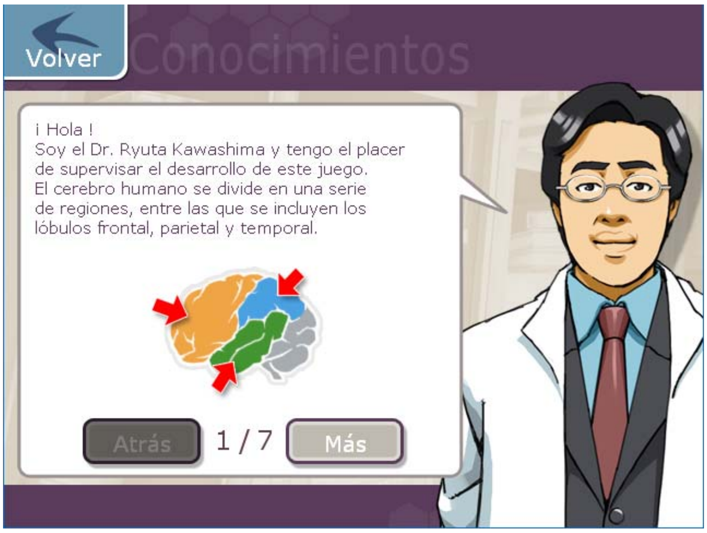

Figure S2. Brain Exercise® video game screenshot

This game allows the specific training of three central regions, the frontal lobe, parietal and temporal (Figure S3).

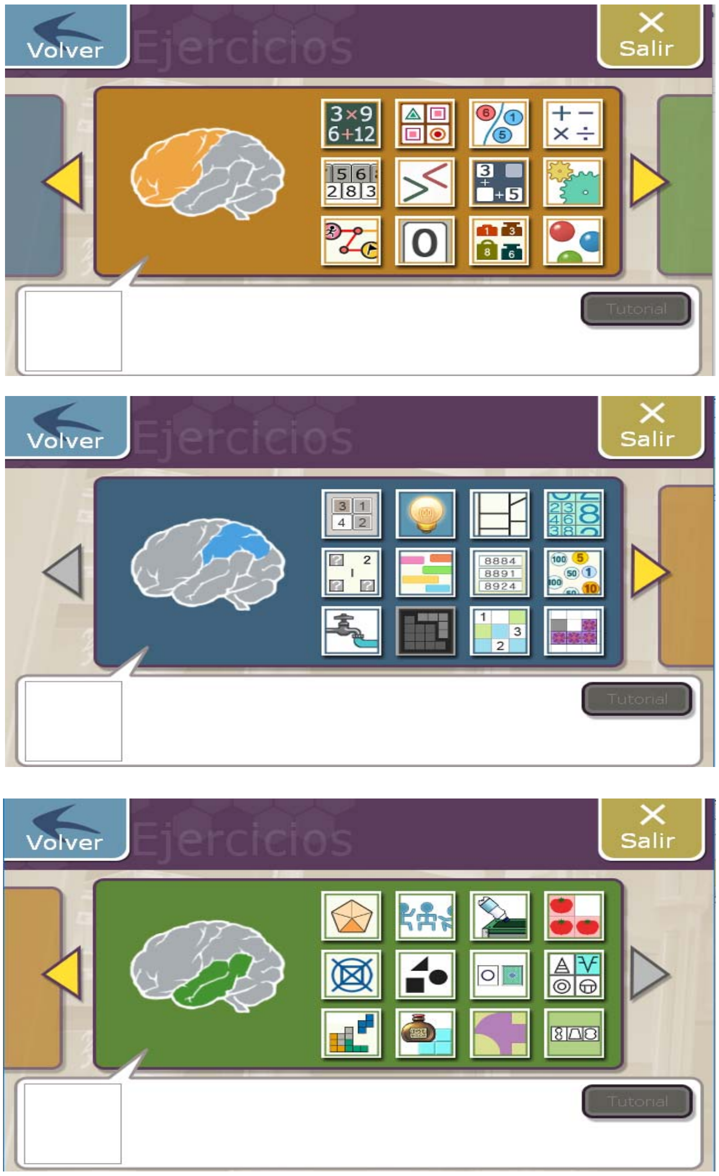

Figure S3. Examples of Brain Exercise® video game Exercises by brain regions

The exercises included in these sessions were based on sorting (e.g., classifying subjects attending to a rule), memory (e.g. remembering the precise location of an object), calculations (e.g. mathematical problems), working memory, logical problems and similar games.

Example of memory exercise:

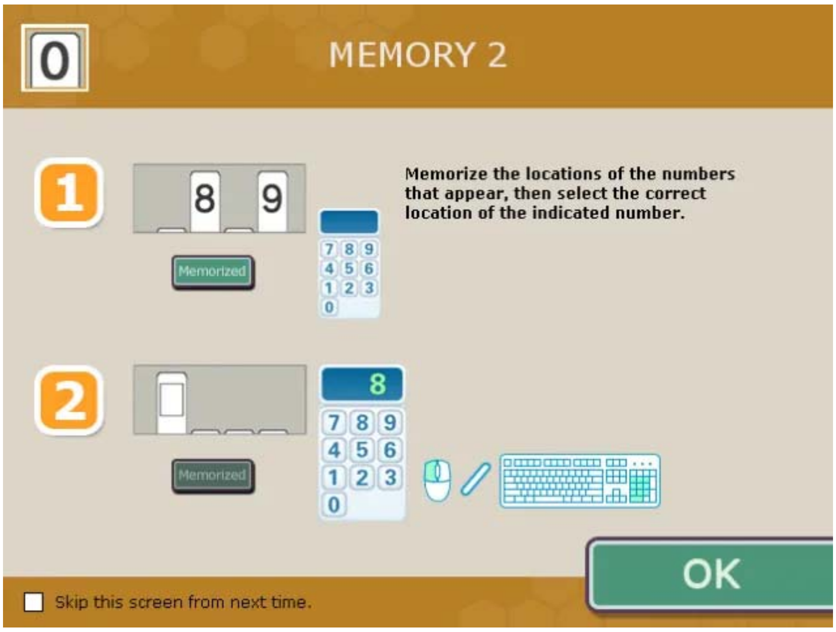

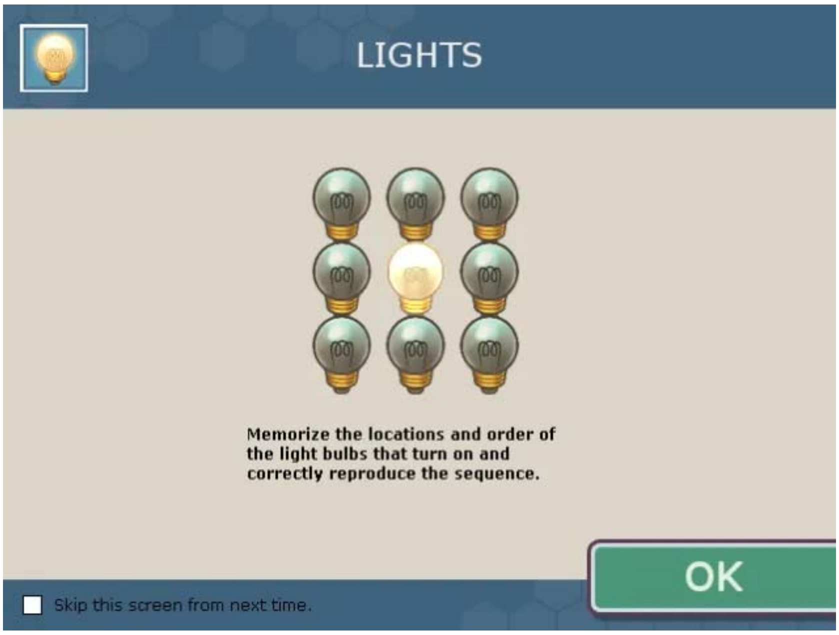

Example of calculation exercise:

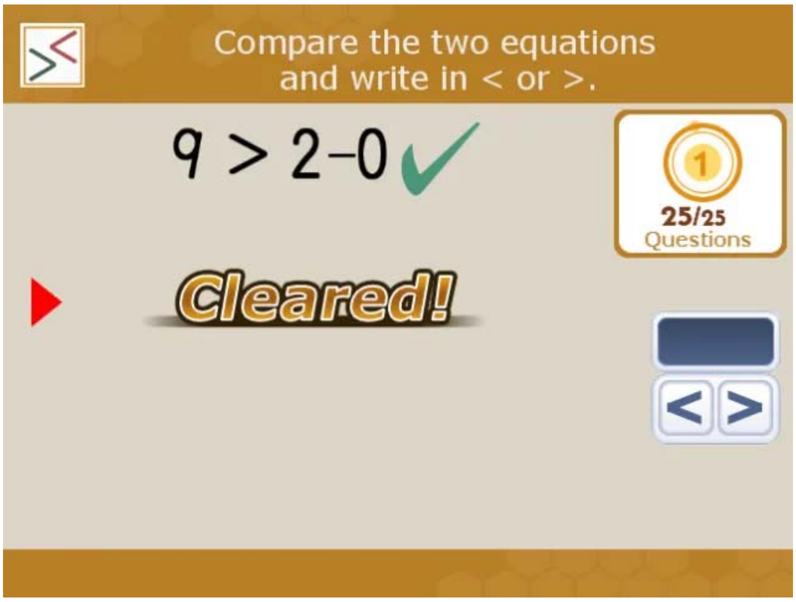

Example of sorting exercise:

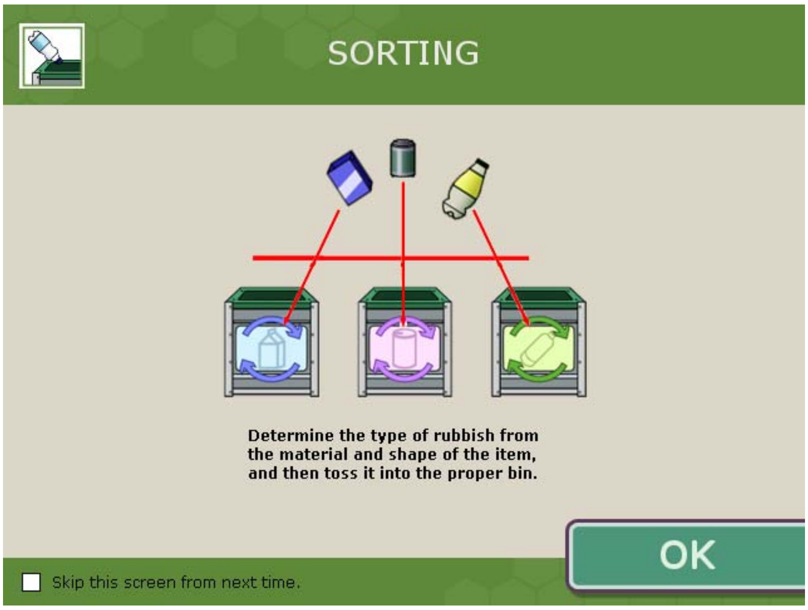

Supplement: Supplementary file 1 [file nutrients-11-00925-s001.zip › Supplementary Information S-1.pdf]
